# Gut-kidney axis regulates renal immune homeostasis via short-chain fatty acids

**DOI:** 10.3389/fnut.2026.1833495

**Published:** 2026-10-30

**Authors:** Chujin Cao, Zimeng Zeng, Rui Zeng

**Affiliations:** 1 Department of Nephrology, Tongji Hospital, Tongji Medical College, Huazhong University of Science and Technology, Wuhan, China; 2 Department of Ophthalmology, Tongji Hospital, Tongji Medical College, Huazhong University of Science and Technology, Wuhan, China; 3 Key Laboratory of Organ Transplantation, Ministry of Education; NHC Key Laboratory of Organ Transplantation; Key Laboratory of Organ Transplantation, Chinese Academy of Medical Sciences, Wuhan, China

**Keywords:** gut-kidney axis, immunity, inflammation, kidney disease, short-chain fatty acids

## Abstract

Short-chain fatty acids (SCFAs) are key gut-derived metabolites, mainly including acetate, propionate, and butyrate, which directly and indirectly interact with or influence immunity. SCFAs regulate renal and systemic immune responses via G protein-coupled receptor (GPR) signaling, inhibition of histone deacetylase (HDAC) activity, and energy metabolism. SCFAs affect not only infiltrated immune cells but also renal resident cells, leading to changes in the renal inflammatory microenvironment. In this review, we summarize the role of SCFAs in systemic and renal immune regulation, by which they may affect the pathogenesis and progression of kidney diseases. Furthermore, we discuss the feasibility of SCFA-targeted intervention as a promising therapeutic strategy for kidney diseases and the challenges faced in clinical application.

## Introduction

Gut microbiota is considered an important factor in regulating host health. This regulation is largely achieved by gut-derived metabolites, which act as a bridge between the gut and extraintestinal organs (1). The proposal of the “gut–kidney” axis concept focuses on the close relationship between gut homeostasis and kidney diseases, and metabolic pathways are key links mediating this crosstalk (2, 3).

Short-chain fatty acids (SCFAs) are the main end metabolites of non-digestible carbohydrate fermentation by gut microbiota, mainly including acetate, propionate, and butyrate. Numerous studies have demonstrated the protective role of SCFAs in various diseases in extraintestinal organs (4–7). Renal damage disrupts gut microbiota homeostasis, characterized by decreased SCFA-producing bacteria and reduced SCFA levels in the circulation and kidney (8). SCFA levels are negatively correlated with kidney disease progression (9). This bidirectional pathophysiological network makes SCFAs crucial renoprotective metabolites in the “gut–kidney” axis and potential targets for breaking the vicious cycle. However, recent studies have reported the pathogenicity of SCFAs (10, 11). The exact roles of SCFAs in kidney diseases and the influencing factors remain unclear.

SCFA receptors are widely expressed on various types of renal resident cells and immune cells. Through downstream signaling pathways, these cells are activated during kidney disease progression. The immunoregulatory and anti-inflammatory effects are important mechanisms of SCFAs, through which they affect the differentiation, migration, and cytokine secretion of immune cells and the kidney inflammatory microenvironment (12, 13). In this review, we searched the PubMed database using “short-chain fatty acids,” “kidney diseases,” and “immunity” as keywords from 2011 to 2026. Original research articles and reviews were included according to PRISMA guidelines, as shown in Figure 1. Based on this literature, we summarized the immunomodulatory function of SCFAs in kidney disease and promising therapies targeting SCFAs, providing a novel perspective for the treatment of kidney diseases based on the “gut–kidney” axis. The innovation of this review is to link SCFAs with immune cells and kidney diseases, distinguish the effects of SCFAs on kidney-infiltrating immune cells versus kidney-resident cells, and attempt to elucidate the general mechanism of SCFA effects on diseases from this tertiary structure.

**Figure 1:**
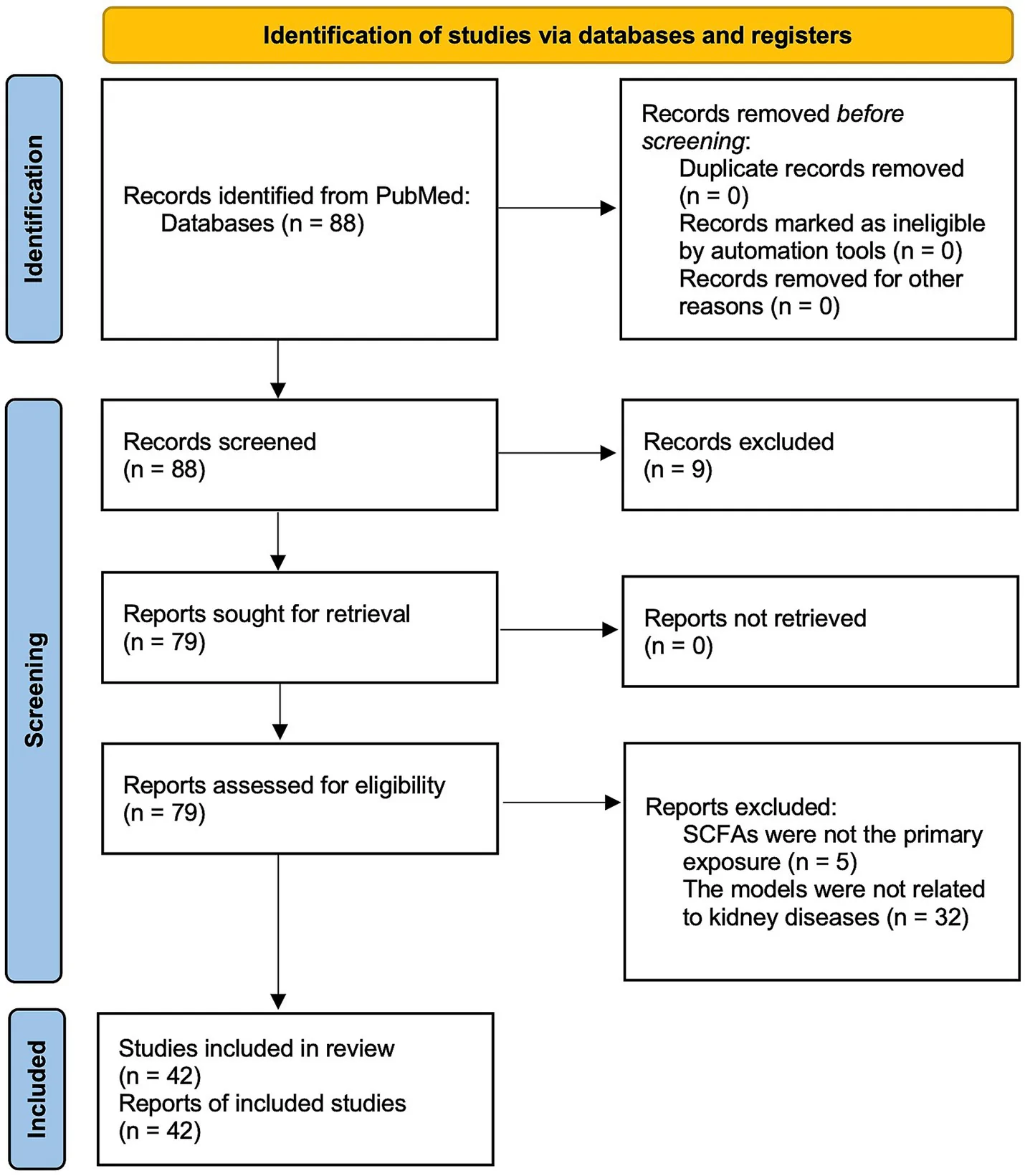
PRISMA flow diagram. The diagram included the identification, screening, eligibility assessment, and inclusion of records for the review in accordance with the PRISMA 2020 statement.

### Pharmacokinetics, receptors and transporters of SCFAs in kidney

SCFAs are used as an energy source by colonocytes or absorbed into the circulation to regulate extraintestinal activities (14). The absorption segment and metabolic location vary among different SCFAs. Acetate is mainly absorbed in the distal colon and enters the circulation to reach extraintestinal organs. Butyrate is absorbed in the proximal colon, and almost all of it is consumed and utilized locally. Propionate is absorbed in all colon segments and metabolized mainly in the liver (15). These metabolic characteristics result in the highest local concentration of SCFAs in the intestine. However, the SCFA concentrations used in *in vitro* studies are difficult to achieve in the circulation and kidney, limiting exogenous SCFA treatment. Pharmacokinetic differences lead to much higher circulating acetate levels than propionate and butyrate, a factor requiring considerable attention in clinical translation. Thus, how to promote SCFA transfer into the circulation and subsequent delivery to the injured kidney is a prerequisite for SCFA application.

SCFAs target effector cells partly by activating G protein-coupled receptors (GPRs). GPR43 and GPR41, also known as free fatty acid receptors 2 and 3 (FFAR2/3), respectively, are fundamental SCFA receptors (16). GPR41 and GPR43 are activated by acetate, propionate, and butyrate. GPR109A, also known as hydroxycarboxylic acid receptor 2 (HCAR2), is another SCFA receptor and is activated only by butyrate among the three main SCFAs (17). GPR41 is expressed on macrophages, and GPR109A is expressed on dendritic cells (DCs). GPR43 is widely distributed on almost all types of immune cells, including neutrophils, macrophages, DCs, T cells, and B cells (12). In addition to immune cells, studies have reported the expression of SCFA-binding GPRs on the surface of renal resident cells. GPR41 is expressed on renal tubular epithelial cells (TECs), vascular endothelial cells (VECs), and the juxtaglomerular apparatus. GPR43 is expressed on podocytes and TECs, and GPR109A is expressed on TECs, podocytes, and VECs. GPR distribution differs among renal tubule segments. GPR41 is distributed throughout renal tubules, including proximal tubules (PTs), distal tubules, and the medullary loop, whereas GPR43 is distributed only on PTs (18). The corresponding relationships among SCFAs, receptors, and target cells are shown in Table 1. SCFAs bind to GPRs and subsequently activate downstream signaling pathways to regulate metabolism, differentiation, expansion, and cytokine secretion of target cells.

**Table 1:** The distribution of SCFA-bound receptors in immune cells and renal resident cells.

Receptor	Ligand	Distribution in immune cells	Distribution in renal resident cells
GPR41	acetate, propionate, butyrate	macrophages	TECs, VECs, juxtaglomerular apparatus
GPR43	acetate, propionate, butyrate	neutrophils, macrophages, DCs, T cells, B cells	TECs, podocytes
GPR109A	butyrate	DCs	TECs, VECs, podocytes

In the colon, SCFAs are taken up into cells via transporters such as SLC26A3, MCT1, MCT4, and SLC4A1/2 (15). However, few studies have mentioned these transporters in the kidney. Our previous research found that Slc18a1 and Slc5a8, SCFA transporters, are expressed on TECs in kidney injury. In addition, SLC5A8 in TECs was decreased in injured kidneys compared with healthy controls, whereas in the fibrotic model, SLC5A8 expression in TECs was unchanged (8). These results provide evidence that SCFAs act on renal parenchymal cells.

### The roles of SCFAs on immune cells in kidney diseases

Various immune cells are regarded as important participants in the pathogenesis of kidney diseases. SCFAs, as immunomodulators, affect many aspects of immune cells. However, direct evidence that SCFAs affect kidney diseases through specific immune cell types is lacking. Thus, whether a definite SCFA/immune cell/kidney disease axis exists deserves more attention. The roles of SCFAs in immune cells related to kidney diseases are shown in Figure 2.

**Figure 2:**
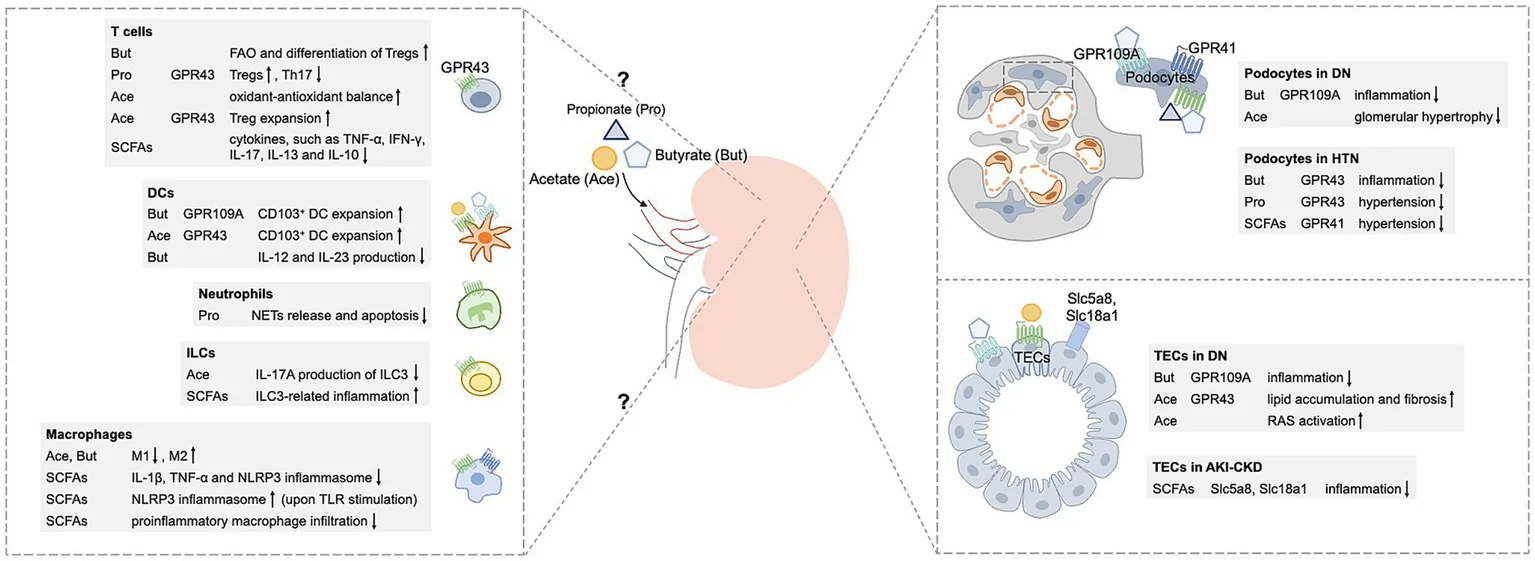
SCFAs regulate immune cells and renal resident cells to participate in kidney diseases. SCFAs target various kinds of immune cells, including DCs, neutrophils, macrophages, ILCs and T cells, which regulates differentiation, expansion, inflammation, and cytokine production of these cells. However, the direct relationship between the regulation of SCFAs on immune cells and the renoprotective effect of SCFAs on kidney diseases requires more exploration. In addition, SCFAs regulate podocytes and TECs via GPRs to directly participate in kidney diseases such as DN, HTN and AKI-CKD. SCFAs, short-chain fatty acids; Ace, acetate; Pro, propionate; But, butyrate; GPR, G protein-coupled receptor; DN, diabetic nephrology; HTN, hypertensive nephropathy; TEC, tubular epithelial cells; AKI, acute kidney injury; CKD, chronic kidney disease; FAO, fatty acid oxidation; Tregs, regulatory T cells; Th, helper T cells; IL, interleukin; TNF-α, tumor necrosis factor-α; IFN-γ, interferon-γ; DCs, dendritic cells; NETs, neutrophil extracellular traps; ILC, innate lymphoid cells; NLRP3, NOD-like receptor family pyrin domain containing 3.

Neutrophil extracellular traps (NETs) are bioactive substances released by activated neutrophils and have been implicated in the pathogenesis of acute kidney injury (AKI) and various causes of chronic kidney disease (CKD) by causing tissue damage and inflammatory infiltration due to their cytotoxicity, thrombogenicity, and autoantigenicity (19). Propionate supplementation inhibited NET release and restored neutrophil apoptosis by inhibiting HDAC1/2/3/6 and enhancing AcH4 acetylation (20). However, SCFAs did not stimulate NET release from human polymorphonuclear neutrophil granulocytes or affect their viability (21). Thus, propionate might improve AKI and CKD by preventing NET formation, but translation to clinical practice requires further studies. Our previous study showed that SCFAs slowed CKD progression by preventing pro-inflammatory macrophage infiltration, providing strong evidence for the benefits of SCFAs in AKI-to-CKD progression through inhibition of macrophage-induced inflammation (8). In animal experiments, supplemental acetate protected allograft mice from rejection and subsequently induced donor-specific tolerance by expanding Tregs in a GPR43-dependent rather than HDAC inhibition-dependent manner (22).

### The roles of SCFAs on renal intrinsic cells

Many studies have demonstrated that SCFAs interact with renal resident cells, including TECs, podocytes, and VECs, through SCFA-binding GPRs on the surface of these cells (14, 23–28), as shown in Figure 2.

Butyrate directly exerted anti-inflammatory responses in podocytes and TECs in diabetic nephropathy (DN) by binding GPR109A (14). The effect of acetate on DN was controversial, possibly related to lipid metabolism. Disruption of cholesterol homeostasis has been shown to promote tubulointerstitial injury in DN. *In vitro*, acetate significantly promoted lipid accumulation in HK-2 cells by activating GPR43 and upregulating the protein expression of HMGR, LDLr, CD36, and CXCL16, leading to a profibrotic effect (23). Acetate was also positively correlated with intrarenal angiotensin II protein expression, promoting RAS activation and contributing to DN-induced kidney injury (24). However, other studies reported that acetate has anti-inflammatory effects and alleviates glomerular hypertrophy, hypercellularity, and interstitial fibrosis (13, 14). SCFAs were found to be decreased in CKD patients with hypertension, and GPRs were also downregulated in hypertensive nephropathy (HTN), with GPR43 being the major receptor decreased in the kidney. GPR43 is expressed on the surface of TECs and podocytes (18, 25). Butyrate ameliorated renal dysfunction and inflammation in HTN via GPR43 signaling and HDAC inhibition, suppressing NF-κB signaling (25–27). Propionate prevented maternal CKD-induced offspring hypertension in early life by increasing renal GPR43 expression (28). Besides GPR43, GPR41 and OLFR78 are other SCFA receptors. GPR41 had lower levels in TECs and VECs in HTN, whereas OLFR78 had higher levels in glomerular cells. Therefore, GPR41 may exert an antihypertensive effect, whereas OLFR78 may have the opposite effect (18). Our previous study identified that TECs expressing SCFA receptors and transporters show increased expression of anti-inflammatory cytokines in ischemia–reperfusion (IR)-induced kidney injury (8).

### The roles of SCFAs on renal inflammation microenvironment

By inhibiting HDAC2, SCFAs enriched the binding of ULK1 promoter sequences to H3K27Ac, causing enhanced autophagy and decreased renal fibrosis in diabetic mice (29). In addition, butyrate reversed miR-7a-5p-mediated targeting of the 3′ UTR of P311 mRNA and suppression of P311 expression, thereby inhibiting transforming growth factor-β1 (TGF-β1) translation and eventually alleviating DN-induced renal fibrosis (30). Butyrate modulated the inflammatory response and attenuated cisplatin-induced kidney injury, characterized by decreased pro-inflammatory factor expression and increased anti-inflammatory factor expression. Butyrate also attenuated p21 and p16 expression, β-galactosidase activity, and ROS production in the kidney, associated with alleviating cisplatin-induced senescence (31). The above evidence is mainly from preclinical experiments. In clinical studies, fecal propionate and butyrate levels in CKD patients gradually decreased with disease progression and were closely associated with serum creatinine (Scr), blood urea nitrogen (BUN), and estimated glomerular filtration rate (eGFR). Mechanistically, propionate and butyrate downregulated TNF-α-triggered expression of genes related to inflammatory processes and immune system activation in TECs (9). Another novel perspective is that SCFAs regulate renal transport proteins, thereby influencing the disposal of uremic toxins (32). Nevertheless, a large gap remains in clinical evidence regarding the effects of SCFAs on kidney diseases.

### Promising therapies for kidney diseases based on SCFA-targeted intervention

In general, SCFAs have a protective role in the progression of kidney diseases, although their effects on immune responses are two-sided. Based on their immunomodulatory function, SCFA-targeted intervention for preventing kidney diseases is considered promising. Several SCFA-based therapeutic strategies exist. One is to control internal SCFA levels by regulating gut microbiota, such as probiotic supplementation. Another is direct intake of exogenous SCFAs. Both approaches have advantages and disadvantages. Dietary or probiotic treatment can alter renal SCFA levels, whereas exogenous intervention is difficult to deliver SCFAs accurately to the kidney without distribution to the circulation or other organs. Probiotic supplementation directly regulates gut microbiota, which greatly diminishes the specificity of SCFA targeting.

#### Clinical evidence for SCFA-based therapies in kidney diseases

SCFAs are produced by dietary fermentation; thus, dietary intervention might significantly affect SCFA levels. A study on how dietary protein restriction affects CKD showed that CKD patients on a low-protein diet had a marked decrease in the abundance of butyrate-producing bacteria, leading to reduced serum SCFA levels compared with patients on a normal-protein diet (33). Supplementation with a fermentable high-fiber diet reduced Scr levels in patients on maintenance hemodialysis, which was related to increased SCFAs (34). Oral administration of probiotics is an effective method to modulate SCFA levels. *Clostridium butyricum* treatment was reported to increase fecal levels of acetate, propionate, and butyrate and urinary levels of acetate and valerate. Increased SCFA levels regulated by *Clostridium butyricum* were associated with decreased inflammation in children with primary nephrotic syndrome (PNS) (35).

#### Preclinical evidence for SCFA-based therapies in kidney diseases

In animal experiments, dietary fiber was reported to improve gut microbiota disorders and promote the expansion of SCFA-producing bacteria, resulting in increased fecal and systemic SCFA levels (13, 22, 36).

*Faecalibacterium prausnitzii* expanded butyrate-producing bacteria and increased butyrate levels in feces and serum, reversing the decreased butyrate induced by CKD with HTN and attenuating renal function deterioration and fibrosis progression (25). *Lacticaseibacilli* supplementation elevated butyrate levels and reduced uremic toxins (37). *Lacticaseibacillus rhamnosus Fmb14*, *Lactobacillus paracasei*, and *Lactococcus cremoris D2022* prevented hyperuricemia by increasing SCFA levels (38–40). Our team discovered that the probiotic *L. casei Zhang* increases SCFA levels not only in serum but also in the kidneys of IR-induced AKI and CKD, but clinical validation has not been performed (8).

Although SCFA-based treatments are promising, many challenges remain for clinical application. First, the selection of SCFAs is confusing because their roles in kidney diseases vary among acetate, propionate, and butyrate. Another problem is whether single SCFAs or a mixture is better. Second, the dosage and timing of SCFA intervention affect their roles. One probiotic formula has been shown to ameliorate renal dysfunction in diabetic kidney disease (DKD) and increase acetate levels *in vitro*. However, that study did not provide direct evidence that the probiotic formula reduces renal damage by increasing acetate levels (41). Two studies found that antibiotic treatment decreased acetate and alleviated renal injury in DKD. However, these studies did not perform acetate intervention in DKD animal experiments or clinical trials; they only observed acetate level alterations and the effect of acetate on HK-2 cells *in vitro* (23, 24). To a great extent, SCFAs exert biological effects depending on their achieved concentration in the kidney. Increased concentrations in serum and stool do not necessarily reflect increased concentrations in the kidney. Exogenous SCFAs have difficulty reaching the kidney specifically without distributing to various organs through the circulation, which diminishes their benefits. Thus, translating experimental dosages, especially those used in preclinical experiments, into real clinical application requires much more effort. Moreover, it should be noted that when exogenous SCFAs are administered, their absorption pathways also differ. Intravenous acetate, but not oral treatment, had a renoprotective role in IR-induced renal injury (42). SCFAs are not without risks, including complications such as ureteritis and hydronephrosis due to T-cell-mediated immune activation (34). Thus, achieving a balance between efficacy and side effects is a major challenge.

## Conclusion

Recently, numerous studies have explored the role of gut-derived SCFAs in kidney diseases via immunoregulatory function. SCFAs affect not only immune cells but also renal resident cells and the inflammatory microenvironment. In the future, more studies are required to confirm how SCFAs regulate renal pathogenic immune cells. Determining the corresponding relationships among SCFAs, immune cells, and kidney diseases will help establish SCFAs as predictive factors and therapeutic targets. The lack of clinical trials limits the translational application of SCFAs. Large-scale, well-designed randomized controlled trials are a top priority.
